# A hybrid metaheuristic framework for epileptic seizure detection in healthcare decision support systems

**DOI:** 10.1186/s42494-025-00238-y

**Published:** 2025-09-01

**Authors:** Indu Dokare, Sudha Gupta

**Affiliations:** 1 Department of Electronics Engineering, K. J. Somaiya School of Engineering (formerly K. J. Somaiya College of Engineering), Somaiya Vidyavihar University, Mumbai, 400077 Maharashtra India; 2 Department of Computer Engineering, Vivekanand Education Society’s Institute of Technology, Mumbai, 400074 Maharashtra India

**Keywords:** Epileptic seizure, EEG signal, Ant colony optimization, Grey wolf optimization, Random forest, Healthcare decision support system

## Abstract

**Background:**

The detection of epileptic seizures is a crucial aspect of epilepsy care, requiring precision and reliability for effective diagnosis and treatment. Seizure detection plays a critical role in healthcare informatics, aiding in the timely diagnosis and management of epilepsy. The use of computational intelligence and optimization techniques has shown significant promise in improving the performance of automated seizure detection systems.

**Methods:**

This research work proposes a novel hybrid approach that combines Ant Colony Optimization (ACO) for feature selection with Gray Wolf Optimization (GWO) to optimize the hyperparameters of a Random Forest (RF) classifier. In this patient-specific seizure detection, ACO effectively reduces the feature set, improving computational efficiency, while GWO ensures optimal RF performance. The method is evaluated on the Children’s Hospital Boston–Massachusetts Institute of Technology (CHB–MIT) and Seina datasets, which include multichannel EEG data from epileptic patients. Performance metrics such as accuracy, sensitivity, and specificity are employed to evaluate the effectiveness of the seizure detection system.

**Results:**

The proposed ACO-GWO-RF pipeline demonstrated excellent performance on the CHB-MIT dataset, with a mean accuracy of 96.70%, mean sensitivity of 92.66%, and mean specificity of 99.24%, outperforming existing approaches. The mean values of accuracy, sensitivity, and specificity obtained using the Seina dataset are 93.01%, 89.82%, and 96.26%, respectively. These improvements highlight the robustness of the hybrid metaheuristic method in handling complex EEG data.

**Conclusions:**

The hybrid metaheuristic approach effectively optimizes the processing and classification of EEG data for seizure detection. Its strong performance across datasets suggests potential for integration into interactive health applications. Furthermore, its patient-specific adaptability makes it a promising tool for personalized epilepsy diagnosis, treatment, and long-term management.

## Background

Healthcare informatics has emerged as a transformative field, integrating advanced computational methods with medical data to enhance diagnostic accuracy and patient care. The advent of artificial intelligence (AI) and machine learning (ML) techniques has further accelerated progress in this domain [1–3], enabling the analysis of complex physiological signals such as electroencephalograms (EEG) [4, 5]. In particular, automated seizure detection using EEG signal has become a critical area of research, addressing the challenges of timely and accurate epilepsy diagnosis. Seizure detection models play a pivotal role in tracking the frequency and duration of seizures. These are crucial parameters in evaluating the effectiveness of anti-seizure medications (ASMs) or other therapeutic interventions [6]. Clinicians can make informed decisions about the patient’s treatment plan using seizure detection models.

Approximately 50 million people globally, spanning all age groups [7], experience epilepsy, one of the most prevalent neurological conditions. A defining characteristic of epilepsy is recurrent seizure activity [8, 9]. A seizure, which results from an abrupt and excessive discharge of electrical activity in a group of brain cells, is an aberrant behavior of the brain that results in loss of awareness, loss of consciousness, and unusual behavior [7, 8]. Most of the time, there is no known reason for epilepsy; however, it can also result from a head injury, stroke, infection of the brain, tumor, or genetic abnormality [7].

**Fig. 1:**
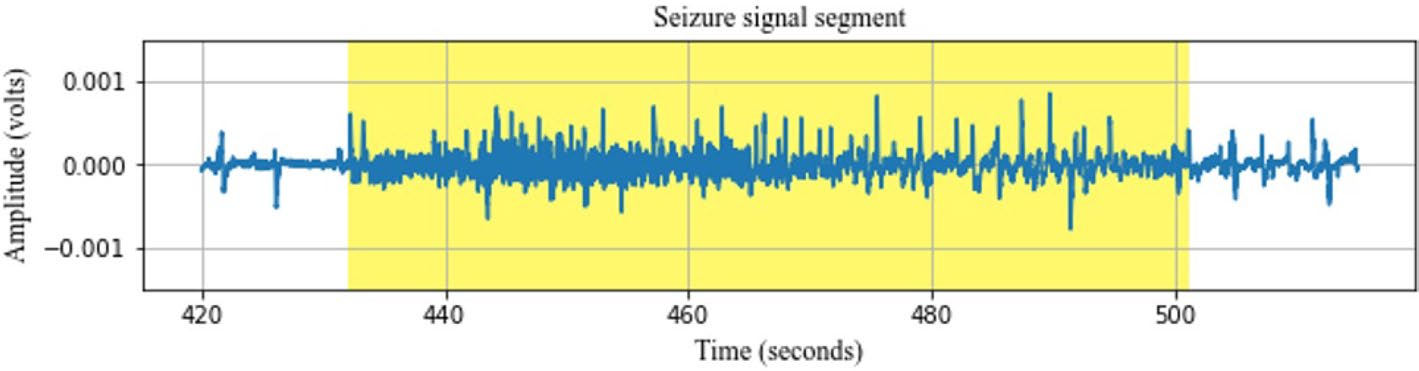
Plot of EEG signal from one channel: The yellow shaded portion shows the seizure signal segment indicating high voltage spikes-and-wave activity

Electroencephalography plays a crucial role in seizure detection [4], capturing electrical activity across various brain regions [9] to identify abnormal patterns associated with seizures. However, analyzing complex multichannel EEG signals for seizure detection presents challenges, particularly in achieving high accuracy, real-time performance, and patient-specific adaptability. In recent years, automated seizure detection has emerged as an essential tool, offering the potential to assist clinicians by streamlining diagnostics and improving patient outcomes through rapid and reliable detection of seizure events [10]. The diagnosis, treatment, and investigation of epilepsy depend heavily on machine learning algorithms and medical signal processing techniques [11]. The process could be automated with the best techniques, simplifying and easing the seizure detection task. The pattern of epileptic seizures is extremely erratic and includes high voltage spikes, spike waves, and complicated spike-wave patterns [12, 13]. Figure 1 shows the plot of the seizure signal segment, which demonstrates distinct spike-and-wave activity, clearly differentiating it from the normal segment.

In EEG-based seizure detection, transforming raw signal data into informative features is critical for enhancing the learning capacity of machine learning and deep learning models. Given the highly dynamic, non-linear, and often chaotic nature of EEG signals [4], researchers have employed a wide spectrum of feature extraction techniques to capture both spatial and temporal dynamics associated with seizure patterns. Time-domain features [14, 15] such as zero-crossing rate [9], signal energy [16], higher-order statistical moments [9, 17] and Hjorth parameters [18] have been frequently used to reflect the signal’s structural properties. In the frequency domain [14, 15], power spectral density, band-specific energy ratios, and spectral entropy [19] are commonly computed to quantify rhythmic brain activity. Time-frequency methods like short-time Fourier transform (STFT) [20], wavelet packet decomposition (WPD), stationary wavelet transform (SWT) [16], and discrete wavelet transforms (DWT) [9, 21] are particularly effective in handling non-stationarity by offering localized information across both time and frequency dimensions.

Furthermore, non-linear and complexity-based features such as the fractal dimension [18, 22], permutation entropy [18, 22], sample entropy [18, 22], fuzzy entropy [22], Lempel-Ziv complexity, and Lyapunov exponents [22] have shown strong discriminative capabilities for distinguishing ictal and interictal segments. In addition to these, more recent studies have explored advanced signal decomposition techniques like dynamic mode decomposition (DMD) [23], which separates complex multichannel EEG signals into spatiotemporal coherent structures and is effective in isolating dominant modes of brain activity during seizures. Similarly, variational mode decomposition (VMD) [24] has been used to extract low-rank representations and intrinsic patterns from EEG, capturing transient and oscillatory behaviour associated with ictal activity. Some approaches also leverage the Hilbert-Huang Transform (HHT) [25] to enhance resolution in time-frequency space. The inclusion of such sophisticated feature extraction methods broadens the representational power of seizure detection models and facilitates the development of systems capable of generalizing across diverse patient conditions. This diverse set of features, when appropriately selected and combined, can significantly enhance the robustness of seizure classification systems.

While the extraction of diverse features enhances the richness of EEG representation, not all extracted features contribute equally to seizure detection. Redundant or irrelevant features can lead to overfitting, increased computational burden, and reduced generalization. Therefore, effective feature selection plays a pivotal role in identifying the most discriminative and informative subset of features. Traditional methods such as recursive feature elimination (RFE) [26], mutual information (MI) [9, 27], Lambda of Wilks (WL) [16], fuzzy C-means clustering [23], and Kolmogorov–Smirnov (K–S) test [28] have been widely used for feature selection in several studies. Similarly, an embedded method such as the least absolute shrinkage and selection operator (LASSO) [29] was also used for feature selection in seizure detection. Recently, metaheuristic algorithms such as genetic algorithm (GA) [30], and binary particle swarm optimization (BPSO) [31] algorithms have gained popularity for their ability to perform global search in high-dimensional feature spaces.

**Fig. 2:**
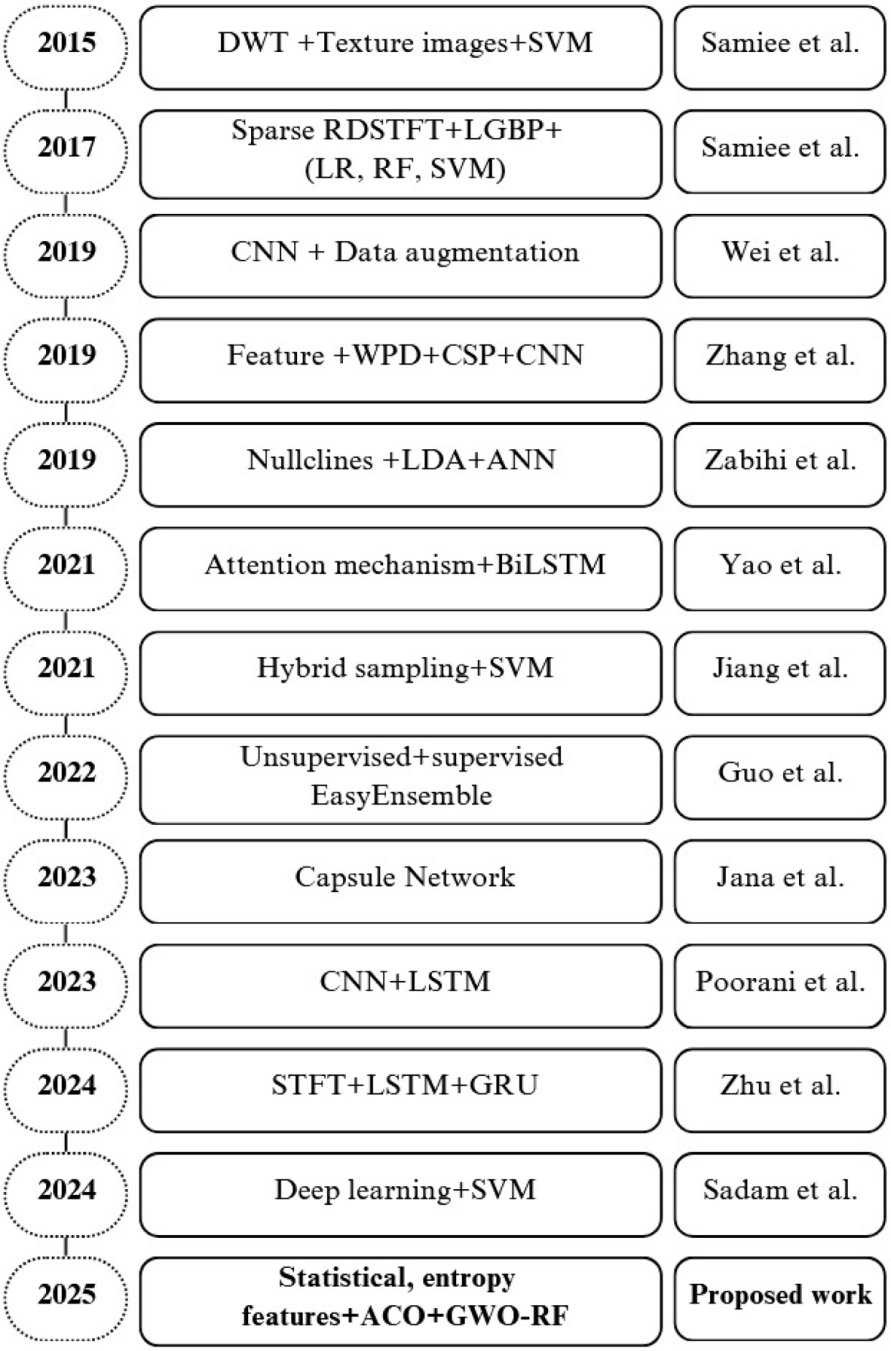
Summarization of existing works

In the past years, numerous researchers have put forth various approaches and models for the investigation and diagnosis of epilepsy detection using multichannel scalp EEG signals [18, 26, 28, 32–34]. Figure 2 presents an overview of existing seizure detection methods based on machine learning and deep learning algorithms.

The patient-specific approach presented [34] has employed a DWT to transform the EEG signal into a 2D space, forming a texture image. The Gray Level Co-occurrence Matrix (GLCM) was used to extract multivariate features from the generated gray-level texture image. The classification of seizure and seizure-free epochs was obtained using support vector machine (SVM), logistic regression (LR), k-nearest neighbor (kNN), Naive Bayes (NB), and random forest (RF) classifiers. Another study [26] has employed parse rational decomposition and Local Gabor Binary Pattern (LGBP) to extract the features. These features were computed for 8 rational components of 23 channels and applied to LR, RF, and linear SVM for classification.

A study employed [28] has demonstrated the use of fuzzy entropy for feature extraction and selected the electrodes having significant variation in entropy during a seizure and non-seizure state. The eigenvectors of these entropy values were fed to the SVM. The investigation [32] has decomposed the segmented EEG signal into four frequency bands using DWT. Three extracted features from each band were classified into seizure and non-seizure segments. This approach was validated using five classifiers named linear discriminant analysis (LDA), kNN, SVM, NB, and classification tree (CT). An approach [35] applied DMD to scalp EEG data for seizure detection by measuring power across EEG frequency bands and using signal curve lengths as features. These features were classified using a random under-sampling boost decision-tree classifier, achieving a sensitivity of 87%. Nonlinear dynamics and the Nullclines concept were proposed in one of the works [36] for feature extraction, and further classified these features using LDA. This work has obtained 91.15% of average sensitivity and 95.16% of average specificity. A hybrid local binary pattern (LBP) and wavelet-based method has been proposed [33] for classifying EEG signals. In this patient-specific approach, a new signal was formed from filtered EEG signals using a local binary pattern. After decomposing this new signal using DWT, univariate and bivariate features were extracted and fed to LDA for classification purposes. Min-max histogram approach for feature extraction [18] was proposed along with time domain features and nonlinear features. Further, after feature selection, these features were applied to SVM and XG Boost classifiers.

The work [37] has utilized an attention mechanism combined with a bidirectional long short-term memory (BiLSTM) network for seizure detection. A feature extraction method combining WPD and common spatial patterns (CSP) was developed, and a convolutional neural network (CNN) was employed for classification purposes [38]. Another study [39] has employed a CNN with data augmentation for seizure detection. The approach [40] based on hybrid sampling and a cost-sensitive SVM has achieved a mean sensitivity of 86.34%. Hybrid frameworks that combine signal decomposition methods with intelligent feature selection mechanisms have demonstrated significant improvements. For instance, using empirical mode decomposition (EMD) to derive intrinsic mode functions, followed by mutual information-based best individual feature selection, has enabled the construction of compact yet informative feature sets, effectively used in multilayer perceptron networks for classification [27]. In another work [2], the proposed method utilized unsupervised learning for preliminary seizure screening and supervised learning with EasyEnsemble for improved detection and class imbalance handling.

A fine-tuned capsule neural network (CapsNet) approach [3] has been employed in one of the recent studies. In another study [41], a convolutional neural network model, AlexNet, was utilized to identify epileptic states by analyzing EEG signals from patients and achieved an accuracy of 98%. A deep learning model integrating CNN and LSTM architecture [42] was employed in a research work. The proposed hybrid model [43] combines deep learning-based feature extraction with a nonlinear SVM classifier for effective implementation, obtaining the 94.48% accuracy. In the latest work [44], EEG time-frequency features were extracted using a short-time Fourier transform and processed by a multidimensional transformer. Long short-term memory network and gated recurrent unit (GRU) further analyzed these features, combined via a gating mechanism for seizure prediction. The work [45] presented a comprehensive seizure detection framework integrating statistical and nonlinear features extracted via DWT after artifact removal using a Butterworth filter. Feature selection was performed using *P*-value-based correlation coefficients and distance correlation methods. A bagged tree-based classifier was then used for classification, and the system also incorporated Explainable AI techniques to enhance transparency. Recent advancements in seizure detection have emphasized the refinement of feature extraction and selection strategies to enhance classification accuracy while minimizing computational overhead. Time-frequency transformation methods like continuous wavelet transform (CWT) are commonly employed to convert EEG signals into scalogram images, allowing deep networks such as CNN to function as automatic feature extractors. These learned features are then typically fed into classifiers like SVM for final binary classification [43].

Many existing methods either rely on exhaustive feature sets without optimization or lack an effective feature selection and parameter-tuning strategy, leading to increased computational complexity and reduced classification performance. In this context, this proposed work introduces a novel approach leveraging metaheuristic algorithms for both feature selection and parameter optimization. Metaheuristic algorithms, known for their ability to explore and exploit complex solution spaces effectively, offer significant advantages in addressing the limitations of conventional optimization techniques. Specifically, this proposed work has utilized ACO for feature selection, ensuring the extraction of the most relevant features while minimizing redundancy. Additionally, grey wolf optimization (GWO) is employed for model parameter optimization, ensuring enhanced detection performance and robustness. By integrating these two metaheuristic techniques, this proposed approach bridges the gap between traditional and advanced methods, providing a more efficient, accurate, and computationally feasible solution for seizure detection.

In recent years, machine learning algorithms have played an increasingly prominent role in developing seizure detection systems. Various classifiers, such as SVM [10, 34, 40, 43, 46] and RF [19, 26] have been widely applied in this domain; their performance heavily depends on the quality of the input features and the optimization of model parameters. Consequently, selecting the most relevant features from the vast multichannel EEG data and tuning the classifier’s hyperparameters is paramount to achieving high detection accuracy.

To address these challenges, the discriminating characteristics of seizure/ictal and non-seizure/inter-ictal signals are estimated by using statistical and entropy-based features [46]. Further, this research work proposes a novel hybrid metaheuristic approach combining ant colony optimization (ACO) for feature selection and GWO for hyperparameter tuning of a classifier. The natural way that ants explore the best paths through a graph serves as the inspiration for ACO, which has been successfully used for feature selection to lower dimensionality while maintaining important information. Grey wolf hunting behavior served as the inspiration for the population-based optimization technique known as GWO, which is employed to fine-tune the parameters of RF to improve their performance in detecting seizures.

This proposed research work aims to bridge the gap between traditional seizure detection methods and the need for optimized, patient-specific solutions that can be applied in real-world healthcare settings. The integration of ACO and GWO not only enhances classification performance but also offers potential applications in developing tailored healthcare solutions for epilepsy management, where reliable and responsive detection systems are crucial.

The main contribution of this proposed work is as follows:

1. Feature extraction and dimensionality reduction: Extraction of statistical and entropy-based features from EEG data to capture critical patterns. ACO is utilized to select the most discriminative features, reducing dimensionality and improving computational efficiency, essential for real-time applications.
2. Optimized classifier performance through GWO: GWO is applied to fine-tune the parameters of the RF classifier, achieving high accuracy and ensuring efficient and reliable seizure detection tailored to individual patients.
3. Good performance on benchmark datasets: The proposed ACO-GWO framework is evaluated on the widely used CHB-MIT and Seina EEG datasets, achieving excellent accuracy, specificity, and sensitivity.

The organization of the next part of this paper goes as follows: the “Methods” section covers a description of the datasets used, pre-processing method, feature selection, and the classifier employed. The results and the comparison with existing works are included in the “Results” section. The interpretation of results is presented in the “Discussion” section, whereas the “Conclusions” section summarizes the key findings and outcomes of the research.

## Methods

**Fig. 3:**
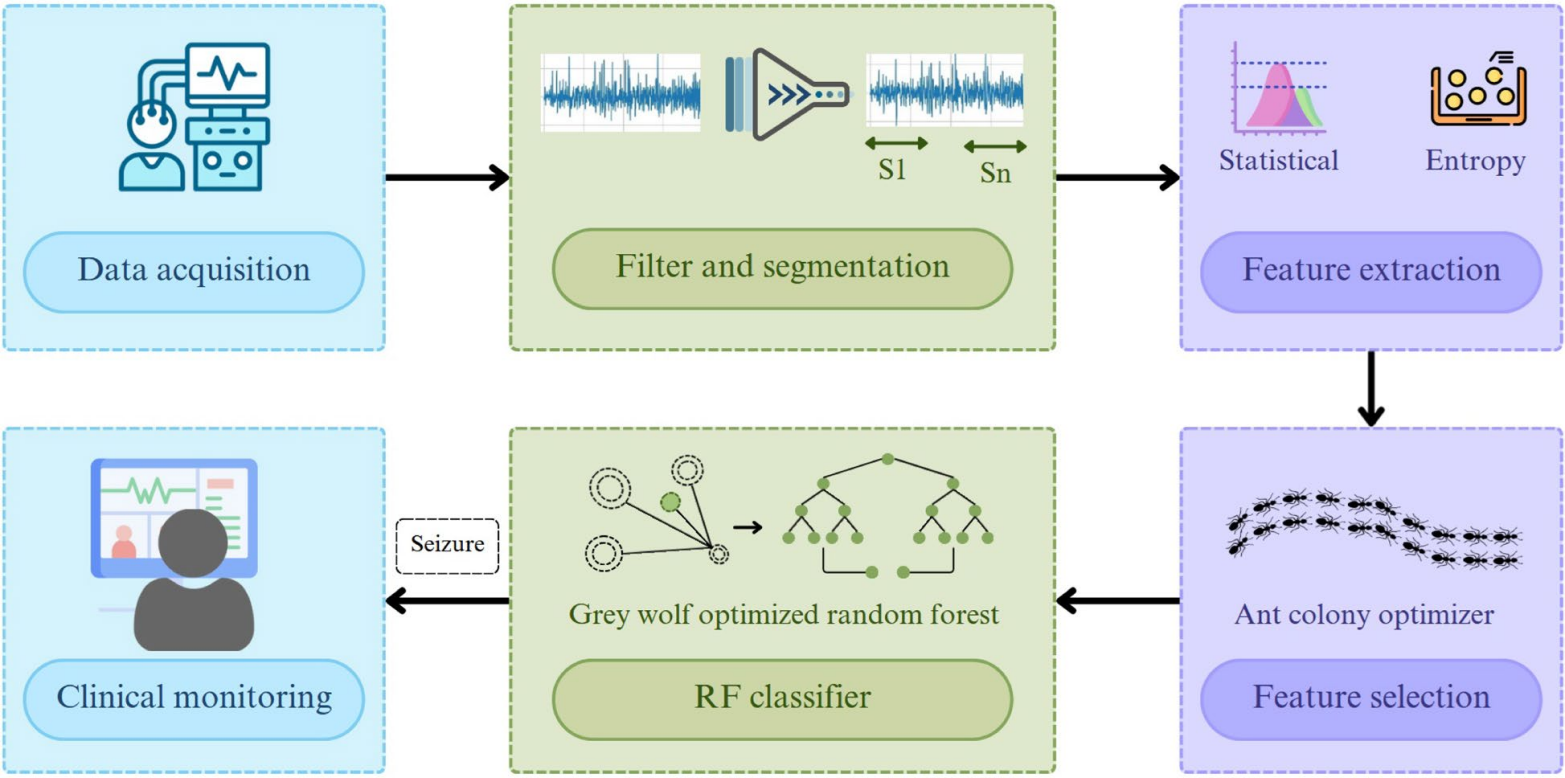
Overview of the proposed framework for seizure detection using the integration of ACO-GWO, illustrating the key stages including preprocessing, feature extraction, feature selection using ACO, and GWO-based hyperparameter optimized RF classifier

The tools and procedures employed in this proposed work for seizure detection are covered in this section. The overview of the workflow used in this proposed work is presented in Fig. 3. The widely known CHB-MIT and Seina multichannel EEG datasets are utilized to assess the proposed hybrid ACO-GWO-RF method.

### Dataset description

This proposed work has been evaluated using two benchmark multichannel EEG datasets: CHB-MIT and Siena, to ensure robustness and generalizability of the developed seizure detection framework.

#### CHB-MIT dataset

The scalp EEG dataset [47, 48], which is accessible online at physionet.org [49, 50], is used in this work for experimentation. The data were gathered from pediatric patients with uncontrollable seizures at Children’s Hospital Boston, using scalp EEG recordings. This dataset was generated and given to Physionet by the Massachusetts Institute of Technology (MIT) and Children’s Hospital Boston (CHB). This multichannel scalp EEG dataset was recorded by the international 10–20 system of EEG electrode locations and nomenclature. The signals were captured with a sampling frequency of 256 Hz with a resolution of 16 bits. It contains a total of 686 recordings, out of which 141 recordings contain 198 seizures in total. The dataset solely includes seizure patients as shown in Table 1. The collection contains some files that show seizure activity, while others do not. The text file specifies the start time and end time of a seizure event. These files include recordings from several channels.

**Table 1:** CHB-MIT EEG dataset used in this proposed work

PT	Gender-age	NS	SD	PID	Gender-age	NS	SD
			(Sec.)				(Sec.)
PT 1	F - 11	7	442	PT 13	F - 3	12	828
PT 2	M - 11	3	172	PT 14	F - 9	8	169
PT 3	F - 14	7	402	PT 15	M - 16	20	1992
PT 4	M - 22	4	378	PT 16	F - 7	10	69
PT 5	F - 7	5	558	PT 17	F - 12	3	293
PT 6	F - 1.5	10	153	PT 18	F - 18	6	317
PT 7	F - 14.5	3	325	PT 19	F - 19	3	236
PT 8	M - 3.5	5	787	PT 20	F - 6	8	294
PT 9	F - 10	4	276	PT 21	F - 13	4	199
PT 10	M - 3	7	447	PT 22	F - 9	3	204
PT 11	F - 12	3	806	PT 23	F - 6	7	424
PT 12	F - 2	40	1475	PT 24	- -	16	511

*PT* Patient, *NS* Number of seizures, *SD* Seizure duration, *Sec.* Seconds, Age in years

**Fig. 4:**
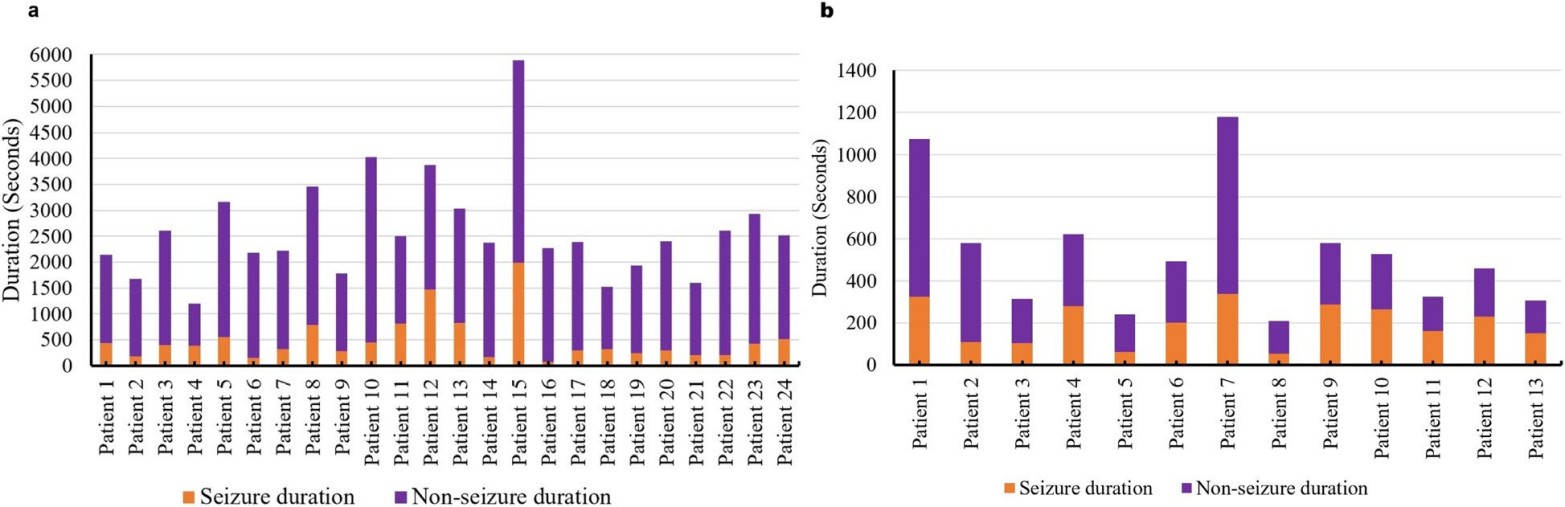
Seizure and non-seizure durations utilized for training the model for each patient in the **a** CHB-MIT dataset and **b** Seina dataset

In this proposed work, 196 seizures are considered for experimentation. The duration of seizure data and non-seizure data considered in this work is depicted in Fig. 4a. The total seizure duration considered is 3 hours, 15 minutes, 57 seconds. The non-seizure data samples are randomly selected from different files recorded at different times for a specific patient.

#### Seina dataset

This dataset contains EEG recordings from epileptic patients, collected at the Unit of Neurology and Neurophysiology, University of Siena. It is publicly available online at physionet.org [51, 52]. The participants were 9 males and 5 females (aged 20–71), monitored through Video-EEG recordings at a sampling rate of 512 Hz. EEG electrodes were positioned according to the international 10–20 system, and data were acquired using EB Neuro and Natus Quantum LTM amplifiers with reusable silver or gold cup electrodes.

In the Siena time series dataset, seizure start and end times are provided in a separate text file, formatted in hours, minutes, and seconds. Detailed information about the patients included in this work is presented in Table 2. The seizure and non-seizure durations used for training the model are shown in Fig. 4b.

**Table 2:** Seina EEG dataset used in this proposed work

PT	Gender-age	NS	SD	PID	Gender-age	NS	SD
			(Sec.)				(Sec.)
PT 1	M - 55	5	325	PT 8	F - 58	1	55
PT 2	M - 54	2	111	PT 9	M - 71	4	290
PT 3	F - 51	3	104	PT 10	F - 34	3	264
PT 4	M - 36	5	282	PT 11	M - 49	4	163
PT 5	F - 20	1	62	PT 12	F - 41	2	230
PT 6	F - 27	3	203	PT 13	M - 42	2	153
PT 7	M - 25	10	338				

*PT* Patient, *NS* Number of seizures, *SD* Seizure duration, *Sec.* Seconds, Age in years

### Data pre-processing

**Fig. 5:**
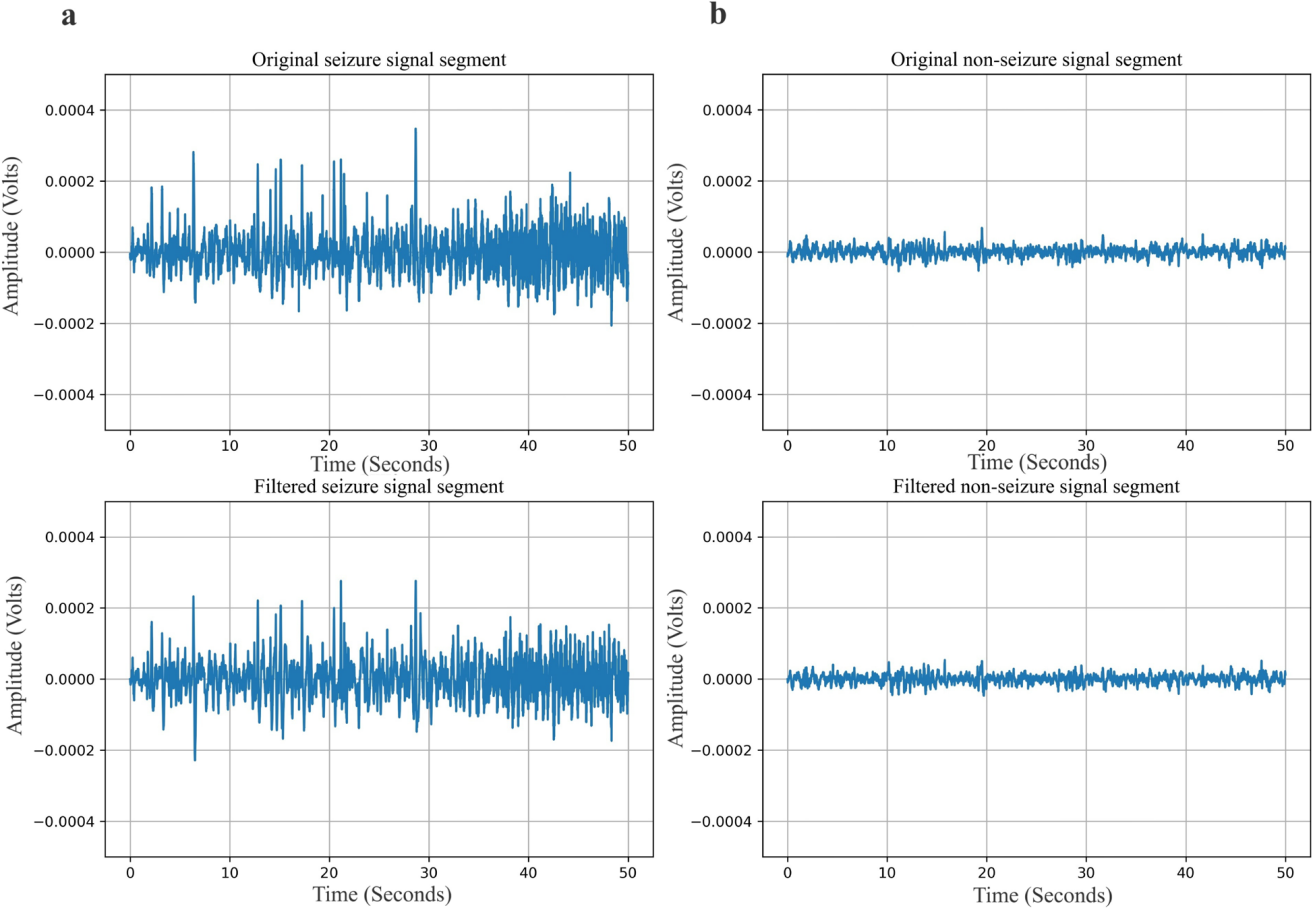
Original and filtered EEG signals from channel FP1–F7 for patient 4 of the CHB-MIT dataset showing **a** seizure and **b** non-seizure segments

The EEG signal contains noise as a result of incorrect recording electrode placement, eye blinks, muscle movements, or 50/60Hz power line interference. As part of the pre-processing step in this work, the EEG signal from every channel is passed through a Butterworth low-pass filter of order 4, having a cut-off frequency of 64 Hz followed by a notch filter. A Butterworth filter is a linear filter that maximally provides a flat response in the passband. The seizure and non-seizure signal segments of channel FP1–F7 for patient 4 of the CHB-MIT dataset before and after the application of the filter are shown in Fig. 5. Since the EEG signal is non-stationary, it is divided into a fixed-size segment to make the signal stationary [4]. For clinical purposes, an epileptic episode often lasts less than 10 seconds.

Many EEG seizure detection techniques typically use segment lengths from 1 to 30 seconds. The segment length employed in various works is 1 second [33], 5 seconds [9], 6 seconds window with non-overlaps [18], 5 seconds window [53], and 4 seconds [32]. This proposed work used a fixed segment length of 4 seconds, containing 1024 sample points for each segment.

### Feature extraction

In order to extract useful information from the raw signal, the main objective of feature extraction is to reduce the enormous dataset into a smaller feature vector. The statistical and entropy-based features [46] of the segmented EEG signal are calculated. Statistical features can capture important information like the shape of the data, distribution, and dispersion, hence can help a model to make accurate detection. The statistical features, like a minimum signal value, maximum signal value, mean, standard deviation, skewness, and kurtosis of the EEG signal, are determined. Entropy is a widely used tool for analysis of the complexity metrics of chaotic time series EEG signals to understand the dynamics of the system based on probability distribution [54]. Hence, the entropy-based features like spectral entropy, sample entropy [1, 55], permutation entropy, and Shannon entropy [1, 55] of the EEG signal segments are determined in this proposed work along with statistical features. Thus, the final feature vector contains six statistical and four entropy-based features, forming a set of a total of ten features. Combining statistical and entropy-based features allows for the identification of complex characteristics like spike and wave patterns and the random nature of seizure activity present in the EEG signals. Ten features are extracted from each segmented EEG signal and are indexed as – 0: Minimum, 1: Maximum, 2: Mean, 3: Standard deviation, 4: Kurtosis, 5: Skewness, 6: Spectral entropy, 7: Sample entropy, 8: Permutation entropy, 9: Shannon entropy. Further, these ten features are fed to the ACO-based feature selection stage.

### Feature selection using ACO

The contribution of each of the ten features varies among patients, meaning not all features are equally important for every individual. Hence, feature selection is essential to identify the most relevant features for each patient, optimizing model accuracy by focusing on features that contribute most to the classification.

Ant colony optimization [56, 57] is a population-based metaheuristic algorithm that draws inspiration from ant foraging behavior. In nature, ants find the shortest path between a food source and their nest by depositing pheromone trails on the ground. Other ants tend to follow these pheromone trails, with shorter paths receiving more pheromone due to frequent traversal. Over time, this collective behavior leads to the discovery of the optimal or near-optimal path. ACO mimics this behavior to solve optimization problems by finding the best solutions through iterative exploration of a solution space. In ACO, artificial pheromone trails represent the desirability of certain paths or solutions. The more pheromones deposited on a path, the more likely that path will be chosen by other ants in future iterations. Ants are agents that traverse a graph representing possible solutions to an optimization problem. Each ant constructs a potential solution by probabilistically choosing components (nodes) based on the amount of pheromone and a heuristic value. ACO finds a balance between exploitation (solidifying already promising solutions) and exploration (exploring new regions of the solution space). This is managed by pheromone evaporation, which prevents early convergence to suboptimal solutions. ACO seeks to maximize an objective function that evaluates the quality of the offered solutions.

This proposed work has employed the ACO for feature selection. In feature selection, the objective function used is the accuracy of the RF model. Pheromone levels are adjusted following each repetition according to how well the ants produced their solutions. Better solutions deposit more pheromones, reinforcing their desirability for future iterations. The ACO algorithm for feature selection is described as follows:

1. Initialization: Initialize the pheromone trails for all potential paths (features) in the search space. Define a number of ants, the evaporation rate, the number of iterations, and the objective function, which is the accuracy of a classifier.
2. Ant solution construction: Each ant starts at a random position (feature). Ants iteratively build solutions by probabilistically selecting the next feature based on the amount of pheromone and a heuristic value. The transition probability for an ant to select feature $'i'$ is influenced by pheromone level ($\tau$) and heuristic value ($\eta$). The probability of selecting feature $'i'$ is given by
  1 $$\begin{aligned} P_{i} = \frac{\tau _i^\alpha \cdot \eta _i^\beta }{\sum _{j \in J} \tau _j^\alpha \cdot \eta _j^\beta } \end{aligned}$$
  where, $P_{i}$ is the probability of selecting feature $'i'$, $\tau _i$ is the pheromone level associated with feature $'i'$, $\eta _i$ is the heuristic information of feature $'i'$, $\alpha$ is the parameter controlling the influence of pheromone levels, $\beta$ is the parameter controlling the influence of heuristic information, $\sum _{j \in J} \tau _j^\alpha \cdot \eta _j^\beta$ is the normalization factor ensuring that the sum of probabilities for all features equals 1. The pheromone level is the amount of pheromone deposited on the feature. The heuristic value is the attractiveness of selecting the feature, based on its importance to seizure detection.
3. Objective function evaluation: Once each ant has constructed a solution (a subset of features), the solution is evaluated using the objective function, which is the accuracy of the RF classifier.
4. Pheromone update: After evaluating all solutions, the pheromone levels are updated. Pheromone is deposited on the features selected by the best-performing ants. The amount of pheromone deposited is proportional to the quality of the solution. At the same time, pheromone evaporation is applied to reduce the pheromone levels of less desirable solutions, allowing for the exploration of new solutions. This helps prevent the algorithm from converging too quickly to a suboptimal solution. The pheromone update rule is given as
  2 $$\begin{aligned} \tau _{i} = (1 - \rho ) \cdot \tau _{i} + \sum \limits _{k=1}^{m} \Delta \tau _{i}^{k} \end{aligned}$$
  Where, $\tau _{i}$ is the pheromone level on feature $'i'$ after updating, $\rho$ is the evaporation rate, controlling the rate at which pheromone evaporates, $\Delta \tau _{i}^{k}$ is the amount of pheromone deposited by ant *k* on feature $'i'$, *m* is the number of ants.
5. Pheromone update and convergence: After multiple iterations, the algorithm refines the subset of features by updating pheromone levels based on the performance of different feature sets. This ensures that only the most relevant features are selected.

In this experimental setup, 5 ants explored possible feature subsets over 10 iterations to identify the optimal combination. Each ant’s feature subset selection was influenced by pheromone levels (importance factor $\alpha$ = 1) and heuristic information (importance factor $\beta$ = 2), which guided them toward promising features. At each iteration, feature subsets were evaluated using RF classifier, and the performance scores influenced pheromone updates, with a 50% evaporation rate ($\rho$ = 0.5) to allow new exploration. This iterative process encouraged convergence toward the best subset of features, thereby improving the accuracy of the classifier for seizure detection. Once the most relevant subset of features is identified, they are utilized as input to the GWO-optimized RF classifier to enhance model performance and ensure effective seizure detection.

### Parameter tuning of RF using GWO

Grey wolf optimization [58] is a metaheuristic optimization algorithm inspired by the social behavior and hunting strategies of grey wolves in nature. GWO has gained prominence for solving complex optimization problems due to its simplicity and effectiveness. The algorithm finds the best answers in various domains by imitating the grey wolf hunting strategy and leadership structure, including engineering, machine learning, and data analysis. In the GWO algorithm, grey wolves are classified into different hierarchical levels: alpha, beta, delta, and omega. This hierarchy is based on their leadership and social roles in the pack. The alpha and beta wolves hold the highest position in the pack, while the omega wolves are the lowest. The alpha wolves are the leaders of the pack. GWO imitates the grey wolf’s hunting tactic of circling and attacking its prey. To get closer to the ideal solution, the wolves, or solutions, adjust their locations in response to the positions of the alpha, beta, and delta wolves. The alpha, beta, and delta wolves’ positions serve as a guide for wolves as they change their places.

RF is a powerful classifier, but its performance is sensitive to hyperparameters. GWO optimizes important parameters that impact RF performance when applied to the RF classifier’s hyperparameter tuning. These parameters include the maximum depth of the trees (max_depth), the number of decision trees (n_estimators), and the minimum number of samples needed to split a node (min_samples_split). The algorithm 1 describes the steps of GWO employed for parameter optimization.

**Algorithm 1:**
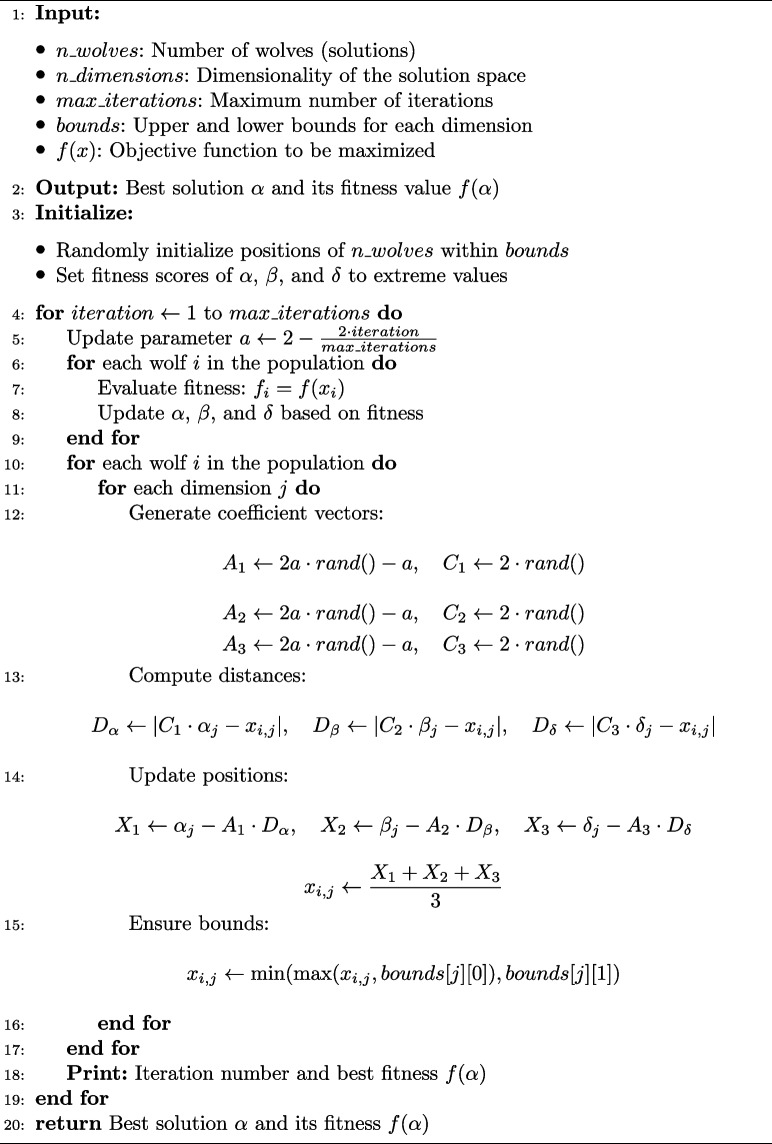
Grey wolf optimization (GWO)

In this proposed work, we have employed a pack of 5 wolves to optimize two parameters of the RF classifier, such as the number of estimators (n_estimators) and the maximum depth (max_depth). The wolves represent potential solutions, where each wolf’s position corresponds to specific values for n_estimators and max_depth. Initially, the positions of the wolves are randomly set within given bounds: n_estimators ranges from 30 to 100, and max_depth from 3 to 20. Over 10 iterations, the wolves update their positions based on the influence of the top three wolves, alpha, beta, and delta, which represent the best-performing solutions. In each iteration, the wolves move closer to the top wolves to refine the solution space.

A control parameter, $\alpha$, which dynamically decreases over iterations to balance exploration and exploitation. During the initial stages, a higher $\alpha$ encourages exploration, enabling the wolves to extensively search the hyperparameter space. As $\alpha$ decreases, the focus shifts towards exploitation, allowing the wolves to refine their search around the best solutions identified so far. The iterative process of position updates and fitness evaluation continues until the wolves’ fitness converges to an ideal solution or the predetermined number of iterations is reached. The position of the alpha wolf represents the most optimal solution. This hierarchical and cooperative behavior helps the wolves converge towards an optimal set of parameters that maximize the model’s cross-validation accuracy, eventually yielding the best combination of n_estimators and max_depth for the RF classifier. This final solution ensures that the RF model achieves improved classification performance, leveraging the integration of metaheuristic optimization with ensemble learning. This approach effectively balances exploration and exploitation, improving the classifier’s performance.

### Metrics for performance evaluation

The performance of the seizure detection model is evaluated by determining the metrics, namely, accuracy, sensitivity, and specificity. Relying on accuracy without properly evaluating the model using other assessment metrics can lead to erroneous predictions when applying a machine learning model to data that hasn’t been observed yet. Hence, this proposed work evaluates the performance of the model using accuracy, sensitivity, and specificity, which are standard metrics derived from the confusion matrix. The confusion matrix provides a comprehensive summary of the classifier’s predictions by categorizing them into four outcomes:

- True positive (TP): Seizure instances correctly identified as seizures.
- True negative (TN): Non-seizure instances correctly identified as non-seizures.
- False positive (FP): Non-seizure instances incorrectly identified as seizures.
- False negative (FN): Seizure instances incorrectly identified as non-seizures.

These values are used to compute the performance metrics as follows:

3 $$\begin{aligned} Accuracy = \frac{TP+TN}{TP+TN+FP+FN} \end{aligned}$$

4 $$\begin{aligned} Sensitivity= \frac{TP}{TP+FN} \end{aligned}$$

5 $$\begin{aligned} Specificity = \frac{TN}{TN+FP} \end{aligned}$$

## Results

The experimental outcomes and comparison with previous work are presented in this section. This includes results of feature selection and analysis of the performance of the proposed patient-specific seizure detection system based on the classification accuracy, sensitivity, and specificity metrics across all patients included in the CHB-MIT and Seina datasets. In accordance with the methodology proposed in our earlier work [46], this proposed work has utilized five selected EEG channels for each patient to ensure efficient processing while retaining critical spatial information relevant to seizure activity. The classifier classifies the data into segments that are seizure/ictal and those that are non-seizure/inter-ictal. Labels ‛1’ and ‛0’ denote the seizure and non-seizure segments, respectively. The selected feature set is split into two subsets: 70% is used for training and 30% is reserved for testing. Five-fold cross-validation is applied during the training phase to prevent overfitting and ensure robust evaluation. A separate model is trained for each patient, and performance metrics, including accuracy, sensitivity, and specificity, are calculated to evaluate its effectiveness.

### Results of feature selection using ACO

**Fig. 6:**
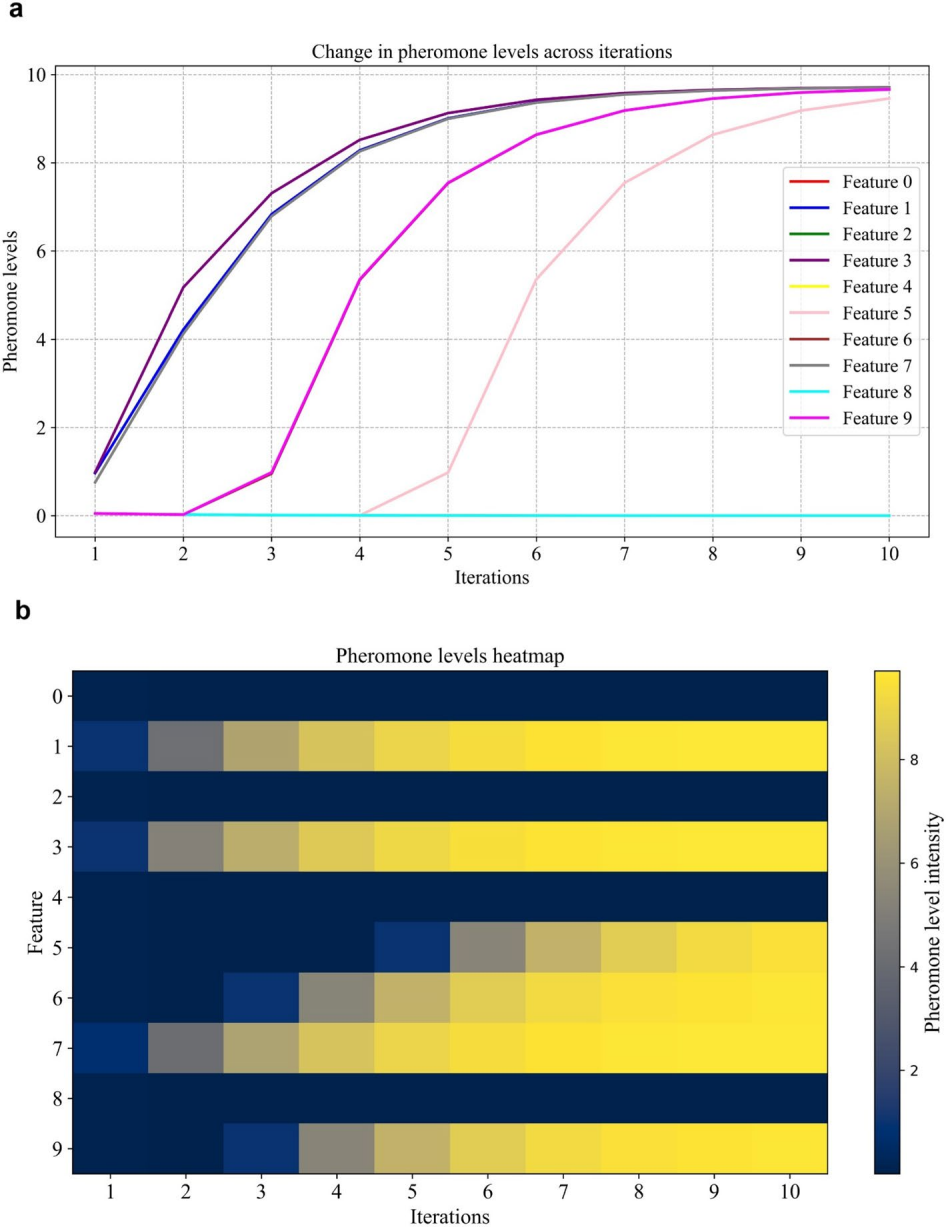
Pheromone level dynamics during the ACO-based feature selection process for patient 8 of the CHB-MIT dataset. **a** Variation in pheromone levels across iterations, **b** Heatmap representing the pheromone level intensity of each feature over iterations (feature indexing– 0: Minimum, 1: Maximum, 2: Mean, 3: Standard deviation, 4: Kurtosis, 5: Skewness, 6: Spectral entropy, 7: Sample entropy, 8: Permutation entropy, 9: Shannon entropy)

After applying ten extracted features to ACO for feature selection, it identifies the most relevant features by iteratively updating pheromone levels on features, guiding the search toward an optimal subset that improves the performance of the RF model. Figure 6a illustrates the evolution of pheromone levels for each feature across multiple iterations during the optimization process for patient 8 of the CHB-MIT dataset. Initially, the pheromone levels are uniformly low across most features, reflecting the algorithm’s initial uncertainty regarding their importance. As iterations progress, certain features, such as features 1, 3, and 7, exhibit consistently increasing pheromone levels, indicating their growing importance in the optimization process. Conversely, features like features 0 and 4 maintain low pheromone levels, suggesting they contribute less to the objective function and are consequently de-prioritized. This demonstrates how the algorithm efficiently identifies relevant features by reinforcing pheromone levels for high-contributing features while reducing those for less important ones, showcasing the adaptiveness of the pheromone update mechanism in feature selection.

**Fig. 7:**
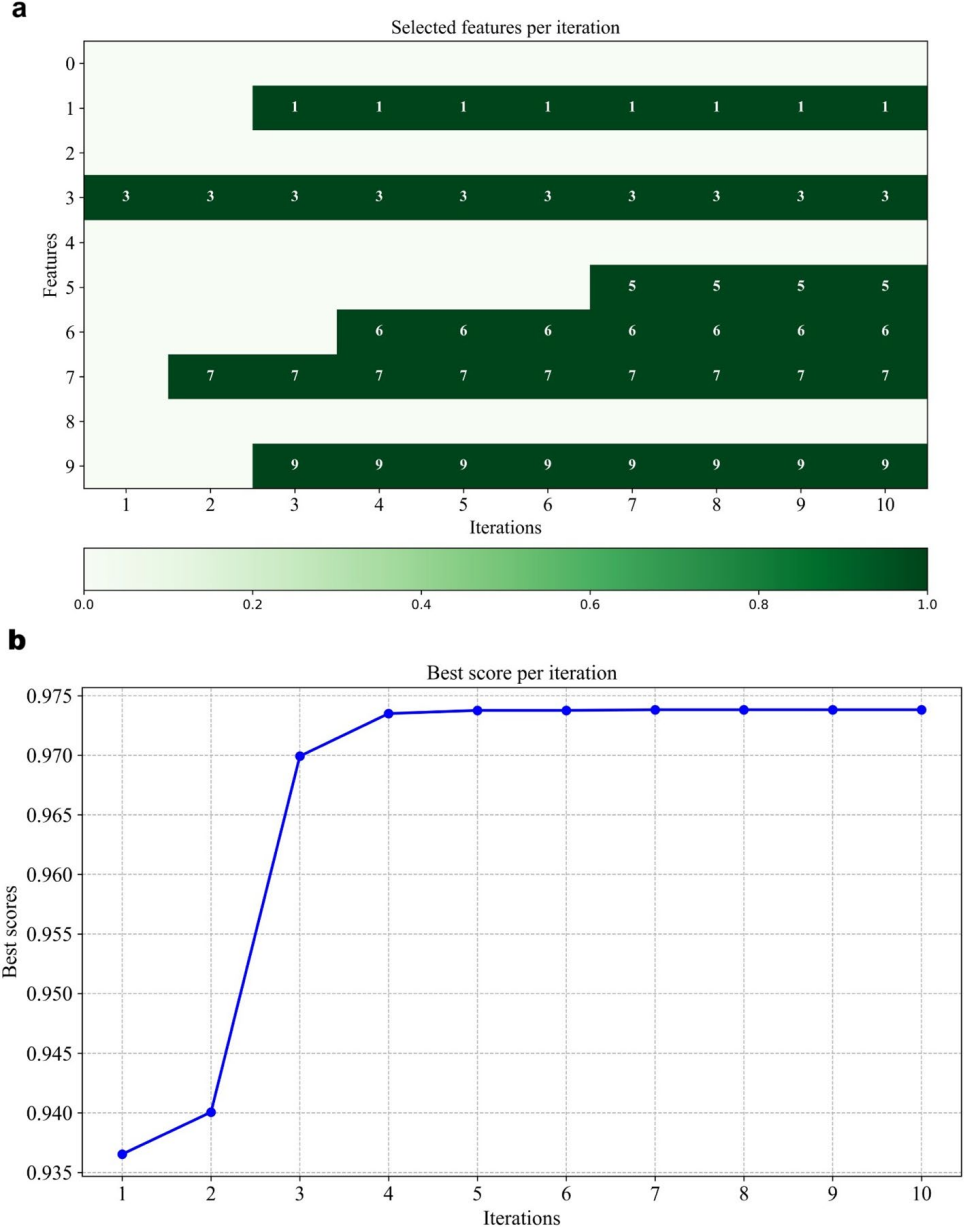
ACO-based feature selection and optimization performance for patient 8 of the CHB-MIT dataset. **a** Selected features per iteration, **b** Best scores across iterations. (feature indexing– 0: Minimum, 1: Maximum, 2: Mean, 3: Standard deviation, 4: Kurtosis, 5: Skewness, 6: Spectral entropy, 7: Sample entropy, 8: Permutation entropy, 9: Shannon entropy)

At iteration 1, the pheromone levels are very low for all features, as the algorithm is just starting to explore. Initially, no feature has a strong preference over the others. This implies that all features are more or less equally likely to be selected based on random probability. It hasn’t yet determined which features are important, so it explores various features. In this case, feature 3 has a higher pheromone level, meaning it was selected more frequently, and therefore, the algorithm believes feature 3 could be important, leading to a best score of 0.9365 as shown in Fig. 7. In iteration 2, the algorithm continues exploring but starts refining its choices. It increases the pheromone level for feature 3, which performed well in the first iteration, signaling a preference for it. Feature 7 is also explored further, as the algorithm evaluates the most beneficial features. It is important to note that feature 1 is not chosen despite having a similar pheromone level to features 3 and 7 in this iteration. This occurs as a result of the algorithm’s selection procedure, which combines a probabilistic decision-making strategy that strikes a balance between exploration and exploitation with pheromone levels. While feature 1’s pheromone level is comparable, the stochastic nature of the algorithm favors other features, like 3 and 7, due to their higher relative probabilities or the algorithm’s inclination to diversify its search in the early stages. However, recognizing the potential importance of feature 1, the algorithm still increases its pheromone level, ensuring it remains a strong candidate for selection in subsequent iterations. This adaptive mechanism helps refine the search space over time while preventing premature convergence.

As the iterations continue, the algorithm becomes more confident in its choices. By iterations 3 and 4, it starts to focus on a smaller subset of features, such as features 1, 3, 7, and 9, because the pheromone levels for these features are higher. Figure 6b shows that the algorithm is exploiting the features it found to be successful in earlier iterations, while still exploring a little in case there is a better feature to find. By the $8^{th}$ iteration, the pheromone levels for features 1, 3, 5, 6, 7, and 9 are high, indicating that the algorithm has exploited these features the most, having found them to be the most beneficial for the task. This shows how ACO efficiently balances exploration (finding new features) and exploitation (focusing on the best ones) to improve results.

Figure 7a shows a binary activation map of features selected during each iteration for patient 8. It illustrates the evolution of selected features across ten iterations using the ACO. It is evident that features such as 1, 3, 6, 7, and 9 are consistently selected from iteration 4 to iteration 10, indicating their strong discriminative power in distinguishing seizure and non-seizure patterns. Other features, like 5, emerged in later iterations, highlighting the progressive refinement of the selection process as the algorithm explores optimal subsets. Figure 7b presents the corresponding best classification score achieved in each iteration. A sharp increase in accuracy is observed from iteration 1 to 3, after which the performance plateaus, suggesting convergence of the optimization process. This indicates that the algorithm efficiently converges to a high-performing feature subset within the initial few iterations, enhancing computational efficiency without compromising accuracy. Features that show persistent activation, such as features 3 and 7, are likely the most critical for the model’s performance. The plot shown in Fig. 7a and b demonstrates that the algorithm successfully converges to a meaningful and stable set of features.

**Fig. 8:**
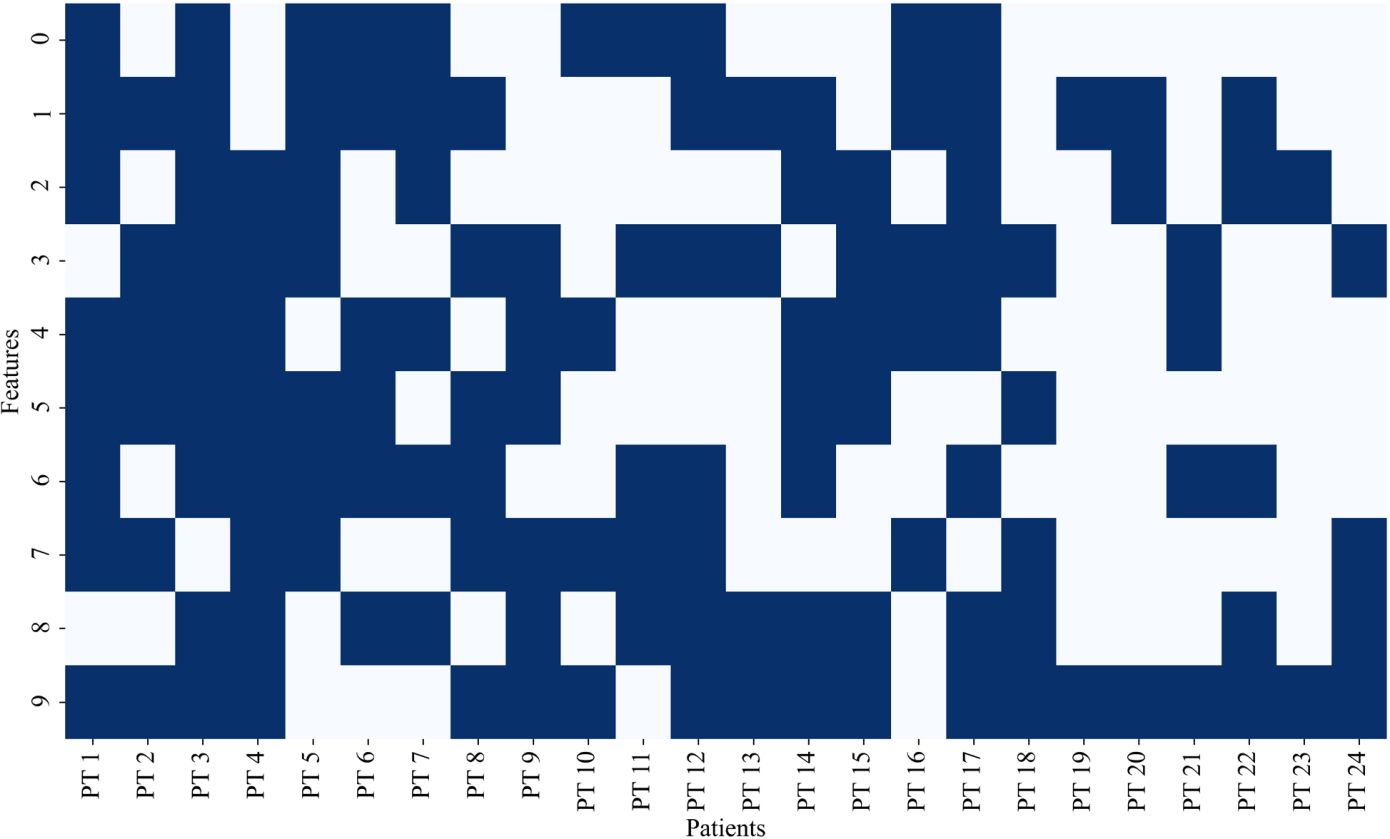
Heatmap showing ACO-based selection of features across 24 patients of the CHB-MIT dataset. The X-axis represents individual patients (PT 1 to PT 24), while the Y-axis denotes different features (0 to 9). The blue shaded cells of the heatmap indicate the selection of specific features for a specific patient. (feature indexing– 0: Minimum, 1: Maximum, 2: Mean, 3: Standard deviation, 4: Kurtosis, 5: Skewness, 6: Spectral entropy, 7: Sample entropy, 8: Permutation entropy, 9: Shannon entropy)

The heatmap depicted in Fig. 8 provides a clear view of the feature selected by ACO across 24 patients of the CHB-MIT dataset, highlighting which features are chosen by the model for each patient. From the heatmap, we observe that feature 9 (Shannon entropy), appears to be the most frequently selected followed by feature 1 (Maximum value), feature 3 (Standard deviation), and feature 8 (Permutation entropy), suggesting that these features are crucial for the model’s ability to detect seizures across various EEG patterns. These features seem critical for the model’s generalizability, making them commonly used in the context of seizure detection across a wide range of patients. On the other hand, some features, such as feature 0 (Minimum value), feature 2 (Mean), and feature 5 (Skewness), are rarely selected with counts 10 and 11, indicating that these may be more patient-specific. Such features may not be as universally informative across all patients or could have less relevance in certain seizure types.

Furthermore, after 10 iterations, the feature selection results stabilized, with no further changes observed in the features chosen by the model. Hence, the feature set selected in iteration 10 is considered final for model evaluation. This suggests that the optimization process has reached a convergence point and further iterations did not yield any new or better performing feature subsets.

**Fig. 9:**
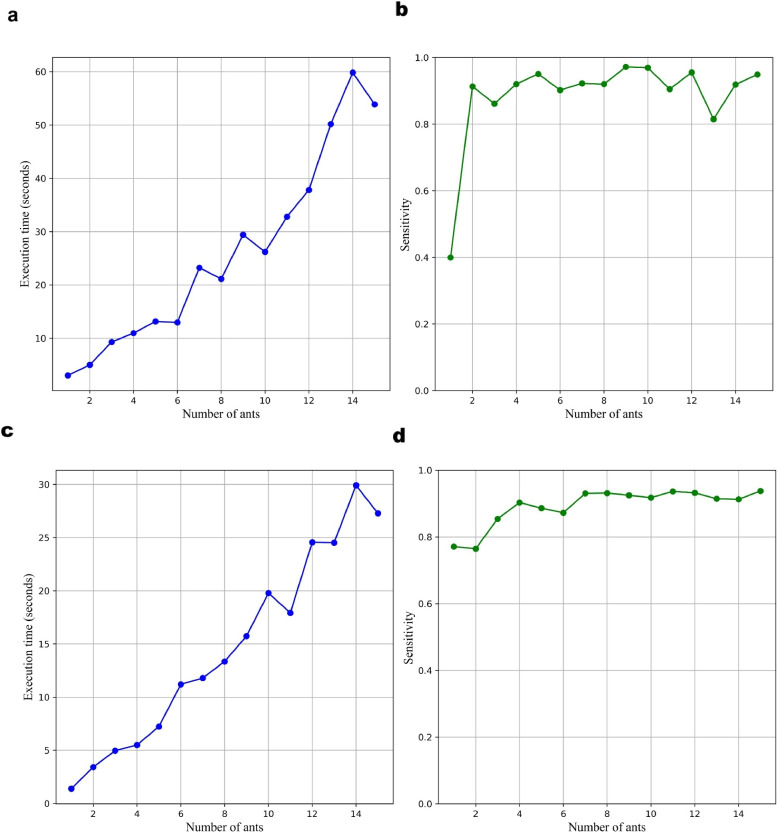
Impact of ant count on execution time and detection sensitivity. This figure shows how changing the number of ants in the ACO-based feature selection algorithm influences (**a**, **c**) execution time and (**b**, **d**) sensitivity for the CHB-MIT (top row) and Siena (bottom row) EEG datasets

To evaluate the influence of the number of ants on the performance of the ACO-based feature selection framework, extensive experimentation is conducted on both the CHB-MIT and Siena EEG datasets. Figure 9a and c represent the execution time for different numbers of ants on the CHB-MIT and Siena datasets, respectively, while Fig. 9b and d show the corresponding variation in sensitivity. Sensitivity serves as a key metric for evaluating seizure detection performance, whereas execution time is assessed to determine the computational efficiency of the method.

From the execution time plots, a consistent upward trend is observed as the number of ants increases. For the CHB-MIT dataset, execution time increases steeply from approximately 3 seconds at 1 ant to nearly 50 seconds at 15 ants, as depicted in Fig. 9a. A similar trend is seen in the Siena dataset, where the time rises from around 2 seconds at 1 ant to nearly 30 seconds at 15 ants, as presented in Fig. 9c. This increase is expected due to the higher computational load and search space evaluation as more ants contribute to the solution search process. These results highlight a critical trade-off between performance and computational efficiency.

On the other hand, as shown in Fig. 9b and d, the sensitivity plots reveal that the performance improves drastically from 1 to 3 ants, then stabilizes beyond 5 ants for both datasets. For instance, in the CHB-MIT dataset, sensitivity jumps from about 0.4 to over 0.9 and remains consistently high, close to 0.9, up to 15 ants, with minor fluctuations. The Siena dataset demonstrates a similar trend, achieving peak sensitivity by around 5 ants and showing negligible gains thereafter. Notably, these timings correspond to processing 671 segments of 4 seconds EEG data from the CHB-MIT dataset and 269 segments of 4 seconds EEG data from the Siena dataset.

These findings suggest that increasing the number of ants beyond five does not substantially enhance sensitivity, while it significantly increases execution time. Therefore, an optimal configuration lies around 5 ants, where high classification performance is achieved with reasonable computational demand. This balance makes the system more efficient and suitable for real-time or resource-constrained environments. Furthermore, it demonstrates the robustness of the ACO-based feature selection method across different datasets, reinforcing its applicability and scalability.

**Fig. 10:**
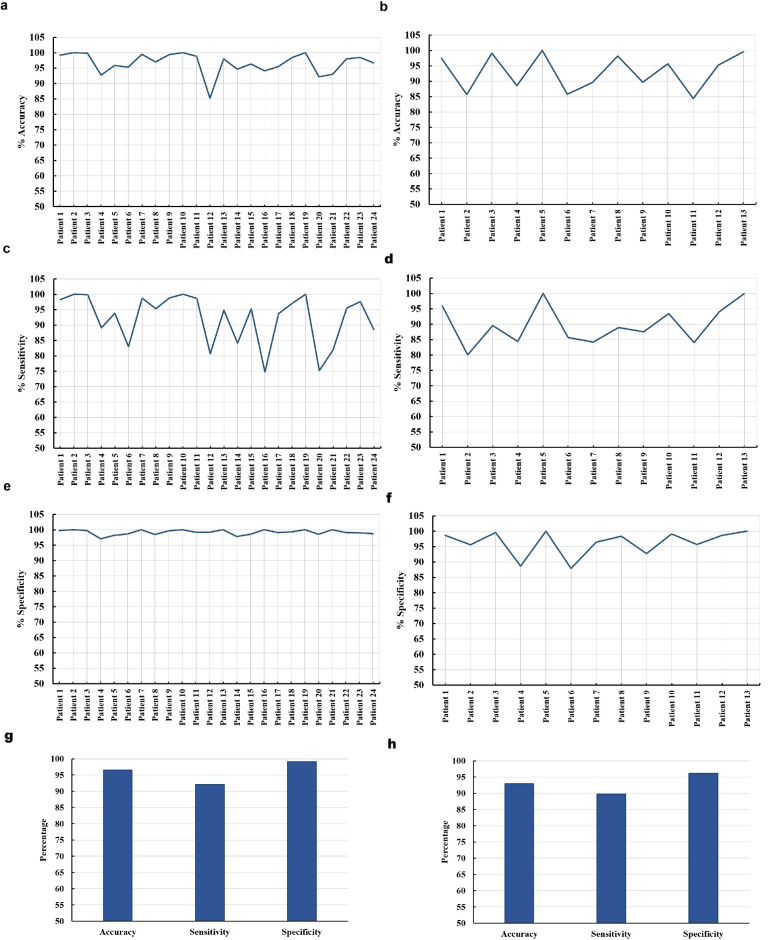
Performance metrics obtained across all patients of the proposed approach on the CHB-MIT and Seina datasets: **a** Accuracy (CHB-MIT), **b** Accuracy (Seina), **c** Sensitivity (CHB-MIT), **d** Sensitivity (Seina), **e** Specificity (CHB-MIT), **f** Specificity (Seina), **g** Average performance across patients (CHB-MIT), and **h** Average performance across patients (Seina)

### Results of a seizure detection

The features selected by the ACO, as illustrated in Fig. 8, are utilized as input to the GWO-optimized RF classifier, enabling patient-specific training and evaluation for improved seizure detection performance. The accuracy for each patient is calculated based on the model’s performance in correctly classifying seizure and non-seizure events. The results varied between patients, reflecting the individualized nature of the EEG signals. The accuracy for each patient, as illustrated in Fig. 10a and b, demonstrates the robustness and generalizability of the proposed ACO-GWO-RF framework. Across both CHB-MIT and Seina datasets, the model consistently achieves high accuracy, typically exceeding 90% for the majority of patients. This indicates that the ACO-based feature selection effectively captures the most informative features while discarding redundant or irrelevant ones. Simultaneously, the GWO-based hyperparameter tuning allows the RF classifier to adapt optimally to the characteristics of each dataset. The stability of accuracy across varied patient profiles suggests strong overall classification capability, making the proposed method reliable for both inter- and intra-subject seizure detection tasks.

Sensitivity gauges how well the classifier can identify seizures within the dataset. As depicted in Fig. 10c and d, the sensitivity of the model measures its ability to correctly identify seizure events. The sensitivity achieved for most of the patients of the CHB-MIT dataset is above 80%. However, slightly lower sensitivity in certain cases, such as patients 16 and 21, can be attributed to factors such as shorter seizure segment durations, higher noise levels, or the very frequent occurrence of seizures. This indicates that the model performs well in detecting seizures for nearly all individuals in the dataset. The sensitivity obtained for the patients of the Seina dataset is above 80%. The proposed method maintains a commendable detection rate in both datasets, validating the model’s competence in identifying seizure events. The feature subsets selected by ACO likely preserve seizure-relevant temporal and frequency-domain characteristics, while the GWO-tuned RF classifier contributes to reducing false negatives. Despite the variability, the overall sensitivity values confirm the model’s clinical relevance in timely seizure detection.

The specificity of the model, which measures its ability to correctly identify non-seizure events, is notably high across all patients of the CHB-MIT dataset, with values consistently above 96% as shown in Fig. 10e. Even the specificity obtained for the patients of the Seina dataset is above 89% as depicted in Fig. 10f. This high specificity indicates that the model is highly effective at correctly classifying non-seizure periods across diverse patients, minimizing false positives and ensuring that normal brain activity is not misclassified as a seizure event. High specificity is crucial in seizure detection, as false alarms can cause unnecessary stress for both patients and healthcare providers, potentially leading to unwarranted medical interventions. This reliability highlights its practicality for real-world clinical seizure detection applications.

The mean performance measures are calculated across all patients to provide a comprehensive evaluation of the model’s effectiveness. The mean values of accuracy, sensitivity, and specificity across all patients of the CHB-MIT dataset obtained are 96.70%, 92.66%, and 99.24%, respectively, as summarized in Fig. 10g. The mean values of accuracy, sensitivity, and specificity across all patients of the Seina dataset obtained are 93.01%, 89.82%, and 96.26%, respectively, as shown in Fig. 10h. The high mean accuracy shows that the model correctly classifies most seizure and non-seizure events across the patient group. The high value of mean sensitivity suggests that the model effectively detects seizure occurrences for a wide range of patients, though slight variability may exist at the individual level. Finally, the high value of mean specificity underscores the model’s strength in avoiding false positives across all patients, reinforcing its potential as a dependable tool for seizure monitoring in diverse patient populations.

**Fig. 11:**
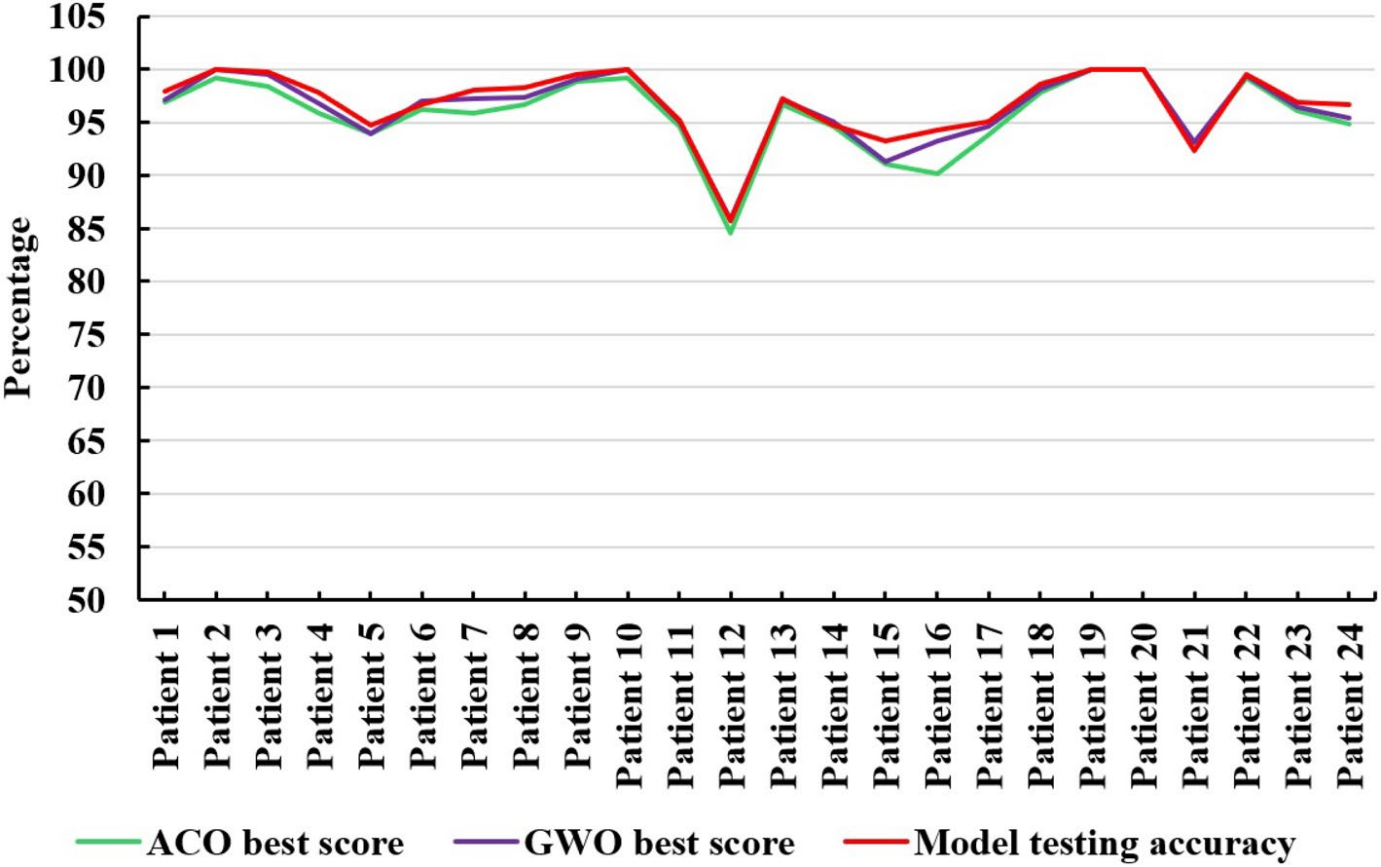
Average best scores across iterations using ACO and GWO optimization techniques and final model testing accuracy using CHB-MIT

The best score achieved during the ACO and GWO stages using the CHB-MIT dataset, as depicted in Fig. 11, reflects the role each optimization technique plays in enhancing model performance. During the ACO stage, the best score represents the highest classification performance achieved by the selected features at each iteration. The ACO stage focuses on optimizing the feature selection, where the best score started from 0.943 and gradually increased, reaching 0.985 by the $4^{th}$ iteration and remaining constant thereafter for patient 8, as shown in Fig. 7b. This signifies the efficiency of ACO in identifying key features relevant to seizure detection. On the other hand, the GWO stage is used for parameter optimization, where it fine-tunes the model’s hyperparameters to achieve the best score. As illustrated in Fig. 11, the plot of the average best scores across patients shows that both ACO and GWO provide considerable improvements in seizure detection accuracy, with GWO achieving higher average scores compared to ACO for most patients.

This suggests that GWO’s nature of exploring the solution space more effectively leads to better optimization, which is reflected in the higher average scores. Additionally, while both techniques yield similar results in terms of performance stability across patients, some individual patients benefit more from one optimization approach over the other. This highlights the importance of selecting an appropriate optimization strategy tailored to the specific characteristics of each patient. The test accuracy of the model consistently outperforms the best scores obtained during the optimization stages using the ACO and GWO techniques. This is due to the continuous improvement of the model through feature selection and hyperparameter optimization, leading to better generalization and overall predictive accuracy compared to the intermediate scores observed during the optimization process.

The proposed patient-dependent seizure detection system demonstrates strong performance across multiple patients from the CHB-MIT and Seina datasets, achieving high accuracy, specificity, and sensitivity. While the system performs well in most cases, the lower performance observed in a few patients from both datasets highlights the need for additional refinement to address patient variability. The system shows great promise for clinical and real-time seizure detection applications, according to the overall results.

### Comparison with existing works

The performance of the proposed method is compared with existing works demonstrated by different authors reported in this paper, as shown in Table 3. The short segment length of 1 second [26] enables fine-grained analysis of rapid signal changes, but leads to increased computational complexity and reduced signal-to-noise ratio. The optimal segment length of 4 seconds is used in this proposed work to reduce computational complexity without sacrificing the accuracy of the analysis for long-term EEG recordings.

The accuracy of this proposed work using the CHB-MIT dataset is 96.70%, which is higher than many of the prior studies with lower accuracies: 92.62% [2], 94.83% [42], and 93.50% [3]. This proposed method using the CHB-MIT dataset demonstrated a 9% improvement in accuracy compared to the approach [37] and an increase of 1% to 3% over the results reported [2, 3, 36, 42, 43]. This improvement highlights the superior performance of this proposed method in accurately detecting seizures.

When comparing specificity using the CHB-MIT dataset, this proposed method achieved 99.24%, which is close to the highest reported specificity of 99.48% by [42]. The specificity achieved by our proposed method is identical to that obtained by [26, 42]. However, our method outperforms [2, 3, 34, 36, 37, 39, 44] demonstrating an improvement in specificity ranging from 1% to 11%. This indicates that the proposed method is highly effective at minimizing false positives, which is a critical aspect in seizure detection applications where false alarms can lead to unnecessary treatments and disruptions.

This proposed method also demonstrates an excellent sensitivity of 92.66% across all patients of the CHB-MIT dataset. An improvement in sensitivity by 1% to 20% is observed compared to prior studies [26, 34, 36, 37, 39, 40] while a decrease of 1% to 5% is observed when compared to [2] across all patients. Unlike previous studies with limited patient cohorts (e.g., 5 patients [3], 8 patients [42], or 18 patients [44]), our approach maintains superior metrics even when compared under matched cohort sizes [3, 42, 44]. This proposed method also exhibits outstanding performance on the Siena dataset, reinforcing its effectiveness and generalizability across different clinical EEG datasets. This work provides a robust seizure detection capability, effectively balancing high sensitivity with minimal false positives.

The comparative analysis highlights the efficacy of the proposed ACO-GWO-RF method in the context of seizure detection. Unlike many existing works that either focus on a single dataset or show imbalanced results across metrics, the proposed method offers a well-rounded performance across both the CHB-MIT and Siena datasets. It demonstrates the ability to deliver consistently high results while maintaining clinical relevance through effective capture of seizure patterns.

This proposed method has achieved over 90% accuracy, sensitivity, and specificity across both datasets, underscoring its robustness and generalizability. This strong performance illustrates the potential of the ACO-GWO-RF approach as a reliable patient-specific solution. The consistent outcomes across varied patient data validate its suitability for clinical environments and reinforce its advantage over several existing approaches reviewed in this proposed work.

**Table 3:** The comparison of performance measures with other reported work. (Performance metrics are expressed as percentages)

Work	NP	NC	LS	Performance metrics		
				ACC	SPC	SEN
Samiee K. et al. (2015) [34]	22	23	1.25	−	97.74	70.19
Samiee K. et al. (2017) [26]	23	23	1	−	99.10	70.40
Wei et al. (2019) [39]	23	23	5	-	92.46	74.08
				-	95.89	72.11
Zhang et al. (2019) [38]	23	-	-	-	-	92.20
Zabihi et al. (2019) [36]	23	-	-	95.11	95.16	91.15
Yao et al. (2021) [37]	23	17	-	87.80	88.30	87.30
Jiang et al. (2021) [40]	22	18	-	-	-	86.34
Guo et al. (2022) [2]	23	18	5	92.62	92.57	95.55
Jana et al. (2023) [3]	5	18	-	93.50	93.90	93.19
Poorani et al. (2023) [42]	8	23	-	94.83	99.48	90.18
Zhu et al. (2024) [44]	18	18	-	-	98.24	97.27
Sadam et al. (2024) [43]	22	23	-	94.48	-	-
**Proposed work: CHB-MIT**	**24**	**5**	**4**	**96.70**	**99.24**	**92.66**
**Proposed work: Seina**	**13**	**5**	**4**	**93.01**	**96.26**	**89.82**

*NP* Number of patients, *NC* Number of channels, *LS* Length of the segment

*ACC* Accuracy, *SEN* Sensitivity, *SPC* Specificity

## Discussion

This proposed work has demonstrated a novel ACO-GWO-RF approach for patient-specific seizure detection using ACO for feature selection and GWO for hyperparameter tuning of RF classifier. Because ACO mimics the behavior of ants seeking the shortest path, it can scan a large area and identify the most important characteristics, making it especially useful for feature selection. ACO is robust in handling high-dimensional data and can effectively reduce the feature set while retaining the most discriminative features, which is crucial in EEG data with many channels and features. This ensures that only the most relevant features for seizure detection are selected, reducing redundancy and improving the classifier’s accuracy. The ACO algorithm effectively selected patient-specific feature subsets, reflecting the variability in EEG patterns among individuals. Each patient’s model converged differently, selecting unique features as shown in Fig. 8 while maintaining high accuracy. This adaptability underscores ACO’s capability to handle inter-patient variability, making it a valuable tool for personalized seizure detection. It is observed that features 1, 3, and 9 are the most frequently selected features, while features 0, 2, and 5 are rarely selected.

The ACO algorithm is particularly suitable for EEG feature selection, as it probabilistically explores combinations of features, preserving diversity and allowing the emergence of globally optimal solutions. To balance the trade-off between computational efficiency and performance, a parameter sensitivity analysis is conducted by varying the number of ants from 1 to 15. It is observed that increasing the ant population improves sensitivity up to a certain threshold, after which the performance stabilizes, particularly after 5 ants in both the CHB-MIT and Siena datasets. However, execution time increases linearly with the number of ants, which presents a practical limitation for real-time applications. An optimal configuration using 5 ants is chosen to ensure computational feasibility without compromising performance.

GWO excels in optimizing the hyperparameters of RF. GWO conducts a global search for ideal parameters, emulating the social structure and hunting techniques of grey wolves to make sure that the classifier is optimized to yield the best results. The adaptive nature of GWO helps overcome the limitations of manual parameter tuning and provides better convergence toward an optimal solution. The adaptive nature of ACO and GWO makes the method particularly suitable for patient-specific seizure detection. ACO can adapt to the specific features of the EEG signal of each patient, while GWO optimizes the classifier’s parameters for better performance on individual patient data. ACO-GWO strikes a better balance, where ACO focuses on exploring diverse feature combinations, and GWO efficiently exploits this information to fine-tune the classifier. As a result, there is a reduced likelihood of becoming trapped in local optima, improving performance overall.

The high specificity of the proposed method ensures that false positives are minimized, making it suitable for clinical applications where avoiding unnecessary interventions is crucial. When compared to other state-of-the-art methods, our approach consistently performs better. This proposed approach has achieved excellent accuracy and specificity, along with relatively high sensitivity for both datasets. This combination of high accuracy, specificity, and competitive sensitivity positions our method as a promising solution for seizure detection, offering a more efficient, reliable, and adaptable model that can be tailored to individual patients. Compared to existing studies [2, 37] employing traditional machine learning models, the proposed framework achieved competitive results, with accuracy exceeding 95% for most patients of CHB-MIT and 90% for most patients of the Seina dataset.

While the proposed framework demonstrates strong overall performance, sensitivity for specific patients, such as patients 6, 12, 14, and 16, is comparatively low. Incorporating data augmentation techniques could help address these challenges by enhancing the training data and improving the model’s robustness. Additionally, the approach requires further validation on larger and more diverse datasets to ensure consistent performance and reliability.

Accurate and timely detection of epileptic seizures is crucial for improving patient care and supporting clinical decision-making. The proposed ACO-GWO-RF framework addresses this need by achieving high sensitivity and specificity, thereby reducing the chances of missed detections and false alarms, an essential aspect of continuous EEG monitoring. This reliability alleviates the burden on clinicians who often manually inspect lengthy EEG recordings. By adapting to patient-specific seizure patterns, the model also aligns with the principles of personalized medicine, offering more individualized assessments. Furthermore, the framework enhances clinical interpretability through the transparent decision-making structure of the ACO and RF algorithms. Systematic analysis of feature selection trends highlights the most influential features contributing to seizure detection, providing neurologically meaningful insights. Overall, the proposed framework stands out as a reliable and adaptable solution for seizure detection, promoting both improved clinical decision support and personalized healthcare outcomes.

## Conclusions

This proposed work highlights the significant potential of metaheuristic optimization methods in healthcare applications, particularly for seizure detection using EEG data. Developing a model for seizure detection is challenging due to the EEG variability among various patients. This work offers a novel method for seizure detection using multichannel EEG data, combining statistical and entropy-based features with metaheuristic optimization techniques. This proposed work has employed ACO for efficient feature selection, reducing complexity while retaining key discriminative information. Additionally, GWO is applied to fine-tune the hyperparameters of the RF classifier, enhancing performance. This proposed work has achieved the mean values of accuracy, sensitivity, and specificity as 96.70%, 92.66%, and 99.24%, respectively, across all patients from the CHB-MIT dataset. The mean values of accuracy, sensitivity, and specificity obtained using the Seina dataset are 93.01%, 89.82%, and 96.26%, respectively.

This proposed methodology, outperforming existing approaches, demonstrates high accuracy, specificity, and sensitivity. The key novelty lies in integrating ACO and GWO, creating an adaptive and robust pipeline tailored to patient-specific seizure patterns. This proposed work offers a promising tool for clinicians, enabling more precise and personalized seizure detection. The proposed ACO-GWO-RF framework can be extended for real-time deployment by optimizing it for lightweight hardware platforms. Future work may also explore the integration of domain-specific interpretability modules to support clinical decision-making and incorporate data augmentation techniques to enhance the diversity of the training data.

## Data Availability

The data used in this work are obtained from publicly available sources: https://physionet.org/content/chbmit/1.0.0/ and https://physionet.org/content/siena-scalp-eeg/1.0.0/.

## Abbreviations

ACO: Ant colony optimization
AI: Artificial intelligence
ASM: Anti-seizure medication
BiLSTM: Bidirectional long short-term memory
BPSO: Binary particle swarm optimization
CapsNet: Capsule neural network
CHB–MIT: Children’s Hospital Boston–Massachusetts Institute of Technology
CNN: Convolutional neural network
CT: Classification tree
CSP: Common spatial patterns
CWT: Continuous wavelet transform
DMD: Dynamic mode decomposition
DWT: Discrete wavelet transform
EEG: Electroencephalogram
EMD: Empirical mode decomposition
FN: False negative
FP: False positive
GA: Genetic algorithm
GLCM: Gray level co-occurrence matrix
GRU: Gated recurrent unit
GWO: Gray wolf optimization
HHT: Hilbert-Huang transform
KNN: K-nearest neighbor
LASSO: Least absolute shrinkage and selection operator
LBP: Local binary pattern
LDA: Linear discriminant analysis
LGBP: Local Gabor binary pattern
LR: Logistic regression
MI: Mutual information
ML: Machine learning
NB: Naive Bayes
RF: Random forest
RFE: Recursive feature elimination
STFT: Short-time Fourier transform
SVM: Support vector machine
SWT: Stationary wavelet transform
TN: True negative
TP: True positive
VMD: Variational mode decomposition
WL: Lambda of Wilks
WPD: Wavelet packet decomposition
